# Long Term Effectiveness of Photodynamic Therapy for CIN Treatment

**DOI:** 10.3390/ph12030107

**Published:** 2019-07-12

**Authors:** Natalia Mayumi Inada, Hilde Harb Buzzá, Marieli Fernanda Martins Leite, Cristina Kurachi, Jose Roberto Trujillo, Cynthia Aparecida de Castro, Fernanda Mansano Carbinatto, Welington Lombardi, Vanderlei Salvador Bagnato

**Affiliations:** 1 São Carlos Institute of Physics, University of Sao Paulo, São Carlos 13566-590, Brazil; 2 Woman Health Ambulatory, Uniara 14801-308, Brazil; 3 TruCytonics LLC, Rockville, MD 20851, USA; 4 Department of Morphology and Pathology, Federal University of São Carlos, São Carlos 13565-905, Brazil

**Keywords:** cervical cancer, cervical intraepithelial neoplasia, human papillomavirus, photodynamic therapy, methyl aminolevulinate

## Abstract

(1) Background: Cervical cancer is the third most commonly diagnosed cancer and the fourth leading cause of cancer death in women worldwide. The highest incidence rates are in Africa, followed by South-Central Asia and South America. According to the Brazilian National Institute of Cancer (INCA), 16,370 new cases of cervical cancer were estimated for each year of the biennium of 2018–2019. About 90% of cervical cancers originate from the malignant progression of cervical intraepithelial neoplasia (CIN) which is classified based on cytohistological characteristics (low- and high-grade lesions). The present study reports the long-term effectiveness of topical photodynamic therapy (PDT) for CIN grades 1 and 2/3 with up to two years of follow up. (2) Methods: A total of 56 patients with CIN 1, ten with CIN 2, and 14 patients for the placebo group were enrolled in this study. (3) Results: 75% (*n* = 42) of CIN 1 patients presented a complete response to PDT and only 23.2% (*n* = 13) of recurrence, progression, and/or lesions remaining two years after PDT. For CIN 2/3 patients, 90% were observed to be cured after one and two years of follow up. (4) Conclusions: PDT presented best results two years after a non-invasive, fast, and low-cost procedure and in comparison with the placebo group, preventing the progression of cervical intraepithelial neoplasia and preserving the cervix.

## 1. Introduction

The human papillomavirus (HPV) is the most common sexually transmitted infection, causing cervical and other cancers such as of the penis, anus, vulva, vagina, and some types of oropharyngeal cancer [1]. Persistent infection with HPV has been identified as a major cause of cervical intraepithelial neoplasia (CIN), the lesion precursor of invasive cervical cancer.

There are more than 120 different types of HPV that can be classified by their risk of causing cervical cancer. HPV types 16, 18, 31, 33, 35, 39, 45, 51, 52, 56, 58, 59, 68, 73, and 82 are considered to be high-risk HPV to cause cancer, and HPV types 6, 11, 42, 43, and 44 are low-risk and are associated with the majority of benign lesions affecting the anogenital areas, such as genital warts [2]. It is also known that between all high-risk HPV types, HPV 16 has been pointed as the most persistent one. Furthermore, other cofactors of high-risk HPV in cervical carcinogenesis may include smoking, use of oral contraceptives for the long-term, number of sex partners, and exposure to other sexually transmitted diseases [2,3]. 

The classification of CIN is based on the cellular features to discriminate dysplasia levels, being CIN 1, mild dysplasia, CIN 2, moderate dysplasia, and CIN 3, severe dysplasia and carcinoma in situ (CIS) [4,5]. However if CIN is diagnosed at an appropriate time before cervical cancer manifestation, it may be cured and cervical cancer prevented [4]. The American Cancer Society’s estimates for cervical cancer in the United States for 2015 are that about 12,900 new cases of invasive cervical cancer will be diagnosed and about 4100 women will die from cervical cancer [6]. According to the Brazilian National Institute of Cancer (INCA) the new cases and related deaths of cervical cancer estimated in Brazil for 2017/2018 were of 16,370 and 5,430, respectively [7]. CIN is reversible lesions in its pre-invasive stage. Most genital HPV infections are transient, and the majority of all cases will cleared spontaneously within 18 months in young women [8]. Conization, cryotherapy, and large loop excision of the transformation zone (LLETZ) are the most common invasive methods and the primary goal in the management of high grade CIN is to prevent the development of invasive cancer by complete surgical destruction of all neoplastic tissue. The conservative methods for CIN treatment and microinvasive cervical cancer are commonly used in young patients [9]. However, the main side effect of invasive procedures in the cervix is preterm delivery early in the third month of pregnancy and the late abortion [10]. Laser treatment has lower rates of hemorrhage but this procedure is not so common due to the high cost [10].

In this scenario, photodynamic therapy (PDT) presents a viable alternative, inexpensive, and non-invasive option for treatment of high and low grade CIN. This is a technique which occurs due to the oxidative effect of reactive oxygen species produced during the reaction between a compound (photosensitizer) and light in a specific wavelength for the photosensitizer excitation [11]. The early detection and diagnosis of premalignant lesions would have the potential to significantly reduce patient mortality.

The use of PDT for treatment of CIN 1/2 is reported in the literature. In a study of 2014, the pro-drug hexaminolevulinate (HAL) was used and the PDT shows a favorable efficacy and safety profile, representing a promising alternative to surgical procedures in patients with CIN 1 [12]. In a review from 2015, the authors said that it is very likely that physicians prescribe topical PDT for CIN treatment as many patients will be keen to consider a non-surgical option [13]. 

The comparative studies of treatment groups (PDT) and control (placebo) groups are important to ensure that the response to clearance is due to photodynamic therapy and not solely because of the light or photosensitizer stimulus of the uterus, separately [14]. In this study we were also able to follow 14 patients for two years from the placebo group, where eight received only irradiation at 80 J/cm^2^ and six patients received only cream containing 20% Methyl aminolevulinate (MAL) in the cervix for one hour. It seems to be a low number of patients in the placebo group, but other authors who work in gynecological PDT and also in the treatment of low- and high-grade CIN report that their placebo groups are in a smaller number than the treatment group and it is known that a significant difference between these two groups is not expected, especially for CIN 1 treatment once most of CIN 1 lesions disappear naturally [15,16].

Our group has been working on this approach since 2008, firstly treating condyloma acuminatum [16]. The alarming statistics of cervical cancer in Brazil led us to start a new clinical project focused on this need. Therefore, in 2012, a new device was developed (CerCa 150 System^®^, MMOptics, São Carlos-SP, Brazil) and in 2013, a new clinical trial was initiated [15], with as a principal goal the prevention of cervical cancer by an early cost-efficient treatment of cervical intraepithelial neoplasia. 

We are reporting a minimally invasive and translational technology with a device approved by the Brazilian Health Regulatory Agency (ANVISA) and by the Mexican Federal Committee for Protection from Sanitary Risks (COFEPRIS). A clinical protocol using topical methyl aminolevulinate (MAL–PDT) was tested in 56 patients with low-grade CIN by Human Papilloma Virus (HPV) infection, and in 10 patients with high-grade CIN 2/3. 

## 2. Results

**Figure 1:**
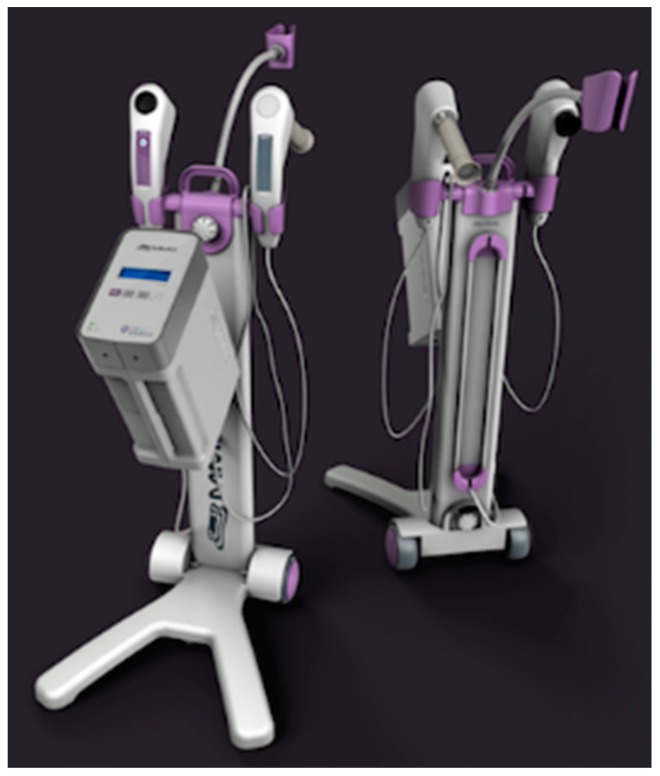
CerCa 150 System^®^ in two different views, showing the front and the back of the device.

CerCa 150 System^®^ development was initiated in 2012, after significant experience of our group with PDT clinical trials for non-melanoma skin cancer [16]. The research with development of devices and clinical trials in partnership with companies and public funding was necessary to achieve these results. The prototype was first used in April 2013 and, after one year, the tests with the new device started, always with the technical advice of Dr. Welington Lombardi, the gynecologist responsible for all patients reported in this study. The final version of the device is shown in Figure 1. 

**Figure 2:**
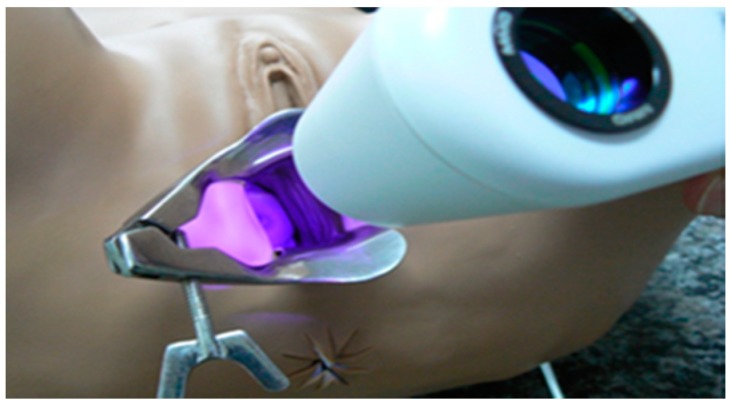
Representative image of the visualization of cervix fluorescence with the CerCa 150 System^®^ using the laser probe emitting at 405 nm.

The intrinsic tissue fluorescence by endogenous fluorophores of the cervix with CIN 1 or 2/3 did not provided a visual discrimination. The photodynamic diagnosis was important to verify protoporphyrin IX (PpIX) production and its consumption by topical medication with MAL at 20% (w/w) in an oil/water cream, and it may help the physician to guarantee the complete PDT (Figure 2). MAL and aminolevulinic acid (ALA) produce the photosensitizer commonly used in PDT, protoporphyrin IX (PpIX), by mitochondria and it is a helpful tool to monitor the PpIX production and consumption during tissue illumination [17,18]. 

Between April 2013 and October 2015, a total of 56 patients with CIN 1 were treated with a single session (1 h of MAL application and 100.8 J/cm^2^ of fluency). The mean age was 25 years old (15–57 year old), and it was an enrolled group where over than 80% of the patients were less than 30 years old. Another group (*n* = 14) received only cervix illumination (*n* = 8) or only MAL cream application (*n* = 6) to analyze the effects of the light and photosensitizer separately. 

A follow-up period of two years was necessary to guarantee the efficacy of PDT in the treatment of CIN 1 against the possibility of recurrence. Patients were then followed up and an annual colposcopy and Pap smear were performed. Among the 56 patients treated, 13 (23.2%) presented treatment failure, where 5.4% (*n* = 3) remained with CIN 1, 8.9% (*n* = 5) had a progression to CIN 2, and another 8.9% (*n* = 5) presented recurrence two years after PDT (Table 1). The dysplasia persistence or progression of the lesion may be due to the inefficacy of the protocol, but it is most probably due to persistence of the HPV infection or reinfection, since these patients are sexually active and remain in the risk group.

There was a complete absence of CIN at two years of follow up in 62.5% of patients (35 lesions). Considering all lesions with positive responses to PDT with follow up of one and two years, the total rate was 75% (42 lesions). Lesion persistence (8 lesions) was 14.3% considered remaining at CIN 1 and lesion recurrence, with only 1.8% abstention. Table 1 summarizes all these results considering the follow up of two years.

The placebo group presented a higher rate of abstention (28.57%) and lesion persistence (14.3%). The complete remission of lesions totalized 57.14%, of which 21.43% presented at the follow up of one year and 35.71%at two years. In a systematic review and meta-analysis by Zhang et al., they concluded that PDT showed more significant results in eradicating premalignant lesions (282 patients) than the placebo group (141 patients) [15].

The distribution of patients’ age shows that most of woman of this study with CIN 1 are above 25 years old, and the study includes girls between 10 and 15 y.o. The reported average age of 25 y.o. for CIN 1 patients added to the fact of later pregnancy shows the need to have techniques capable of preserving the cervix of women who are still going to get pregnant, and also of preventing lesion progression.

The high-grade CIN treatment occurred between April 2015 and September 2016, with a total of 10 patients with CIN 2/3 treated with two sessions, using the MAL cream application with three hours of incubation and a total delivery light dose of 180 J/cm^2^. These CIN 2/3 patients had the mean age of 30 y.o. (18–49 y.o.). In Brazil, in cases of high-grade lesions, the LEEP (loop electrosurgical excision procedure) is established by INCA, and in this study, a less profound cervix removal was realized 60 days after the second PDT session.

Considering all lesions with positive responses after two PDT sessions and 60 days after the second PDT procedure, 40% (*n* = 4 lesions) regressed to CIN 1 or just had chronic cervicitis, but 60% (*n* = 6 lesions) showed high-grade lesions in the histopathological analysis of part of the affected cervix. However, after the follow up of one and two years, a total cure rate of 90% (*n* = 9 lesions) remission was observed and only 10% (1 case) returned with high-grade lesions (Table 1). 

## 3. Discussion

**Figure 3:**
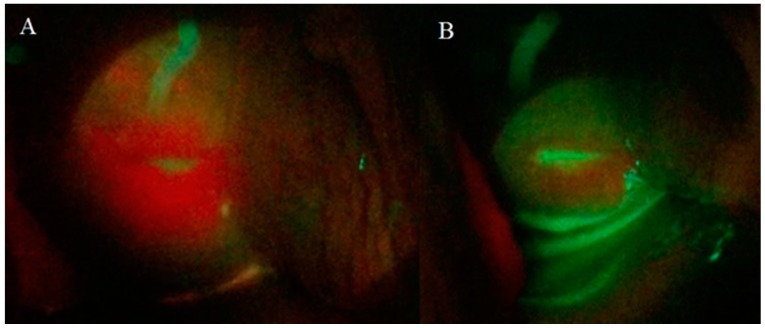
Cervix images of a patient with CIN 1 obtained with CerCa 150 Systems^®^ coupled with Sony Xperia^®^. Panel **A**: protoporphyrin IX (PpIX) fluorescence in the cervix and Panel **B**: cervix fluorescence after PDT (LED 630 nm, 80 mw/cm^2^, with a total dose of 100 J/cm^2^).

With the use of a fluorescence probe, an intense PpIX fluorescence homogenously was observed at the surface mucosa of the cervix tissue, but it was concentrated in the squamocolumnar junction (SCJ) shown in Figure 3A. PpIX photobleaching was observed in all patients immediately after the cervix PDT illumination (Figure 3B). These results indicate a great tool to follow the PDT steps and to ensure for the physician that a complete treatment was performed.

**Figure 4:**
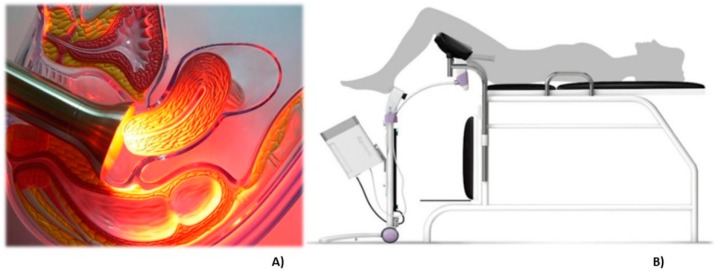
The representative image of the treatment of cervix by photodynamic therapy with the CerCa 150 System^®^ using a LED tip emitting at 630 nm. Panel **A**: transversal view of the cervix during illumination. Panel **B**: an illustration demonstrating the clinical procedure.

The probe for PDT irradiation contains LEDs emitting at 630 nm, a wavelength indicated for PpIX activation. An acrylic tube is used to guarantee the homogenous light distribution and a stainless steel instrument that can be autoclaved. The procedure is comfortable for the patients and during the total time of illumination, they did not report any discomfort or pain. In some cases, a mild colic was reported. In Figure 4A, there is an illustration of the irradiation probe in direct contact with the cervix and at panel B, a drawing of the clinical setup is shown. 

According to the literature, CIN 1 lesions have a natural regression. However, Östor et al., in 1993, in a review of the natural history of cervical intraepithelial neoplasia, reported the regression rates of CIN 1 as 57% (the same rate of the placebo group of the present study), persistence as 32%, and progression of 11% [19]. Because of this high percentage of natural regression, the patient with CIN 1 does not usually receive any type of treatment procedure and the examination is repeated after one year, when the patients return. Of this percentage, then, there is a 43% of probability that this lesion remains there or, even worse, progress to a higher degree dysplasia, potentially resulting in a more complex patient management and higher cost treatment. There is also the possibility of the patient does not return, as shown by the high rate of no follow up of our placebo group.

Hillemanns et al. tested the effectiveness of topical HAL in 47 CIN 1 patients who underwent PDT and only 12 other patients in the placebo group who received only one vaginal suppository containing the pro-drug HAL. They observed that the HAL PDT response in the CIN 1 population was not significantly different to that of the placebo group, due to a high rate of spontaneous regression in the CIN 1 population. This was probably caused by the inclusion of oncogenic HPV-negative patients [13].

**Figure 5:**
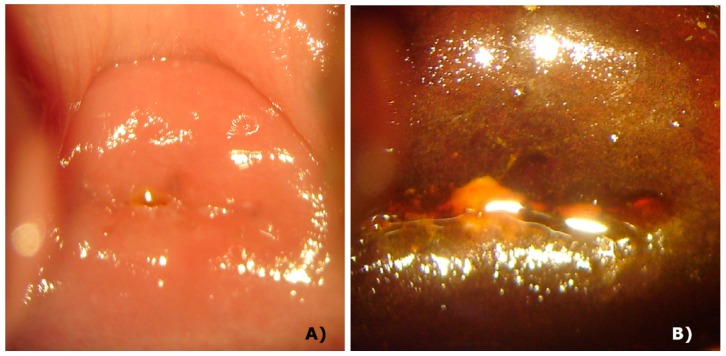
Cervix 2.5 years after PDT. Panel **A**: white light image, showing the integrity of tissue. Panel **B**: white light image after colposcopy, with no damage.

Although some countries indicate only the follow-up of the patient without performing any procedure as a standard procedure, if the physician wants to perform some procedure, she/he has only the option of conization. Among the side effects of this procedure, there is the loss of cervical integrity, increasing the possibility of premature birth, the closure of cervical canal making menstruation impossible, and increasing the possibility of a carcinoma and infections [20,21]. On the other hand, photodynamic therapy has a great tissue repair rate and the cervix maintains its anatomical and mechanical characteristics, without clinical alteration of function and physiology. Figure 5 shows the integrity of the cervix of a patient after 2 years and 9 months of PDT procedures and it is clear there is no damage of the tissue. After the colposcopy, the same cervix without lesions is clearly shown in this figure. Some patients got pregnant during the follow-up, showing the integrity of the cervix.

According to the Brazilian National Institute of Cancer (INCA), the age range for screening for cervical cancer is 25 to 64 years old and once positive for CIN 1, they should repeat the cytology in three years and there will be only some intervention if CIN 1 persists or there is progression of the lesion to 2/3. Due to the territorial dimension and faulty distribution of medical specialists throughout Brazil, this long follow-up time can mean the patient’s non-return and the evolution of the lesion to a more serious condition [7].

Considering the cervical intraepithelial neoplasia of high-grade CIN 2/3, the standard treatment is the transformation zone removal or conization. However, complications involving that treatment are described by diverse authors and the main concern is due to the reproductive future, since the disease occurs more frequently among young women [4]. In our study, the histopathological analysis of LEEP samples 60 days after PDT indicated the presence of high-grade CIN (2/3) in 60% of all patients treated with PDT. These findings differ in comparison with Barnett et al. [22], who achieved an eradication rate of 66% with PDT and placental group (LEEP) with the LEEP three months after PDT. This may have happened because two months may have been a small time to see tissue changes from injury reduction. In addition, there is a possibility of disparity in histological findings between punch biopsies and samples obtained by LEEP [23].

With the follow-up of one and two years, the eradication rate of 90% with PDT was observed, and with only one case of recurrence of CIN 3 (Table 1). Zhu et al. showed that of the 238 patients, 211 (88.7%) patients remained free of persistence/recurrence, while 27 (11.3%) experienced persistence/recurrence, in a median follow-up period of 25 months [24]. In another study, the authors concluded that women who have undergone excisional treatment for high-grade CIN indicate a very low risk for recurrent disease and potentially negligible risk for invasive cancer, provided that a strict and vigorous follow-up is offered after treatment [25]. Therefore, the PDT associated with LEEP can decrease recurrence rate and, in addition, the literature shows that this treatment can stimulate the patient’s innate immune system, helping to clear HPV infections [26], whereas a cohort study of 201 patients performed with patients in Brazil showed a total cure rate in 80% (*n* = 161) of all patients who underwent conization with LEEP of the high-grade cervical intraepithelial lesions, followed for an average of two years. In the results presented here, the 90% of cure after long term follow up is may be due to both the reduction of viral load, and associated with the LEEP procedure.

The variables that initially presented statistical significance as indicators of risk for recurrence were: number of partners, seropositivity, cone margins, and glandular involvement. The simultaneous occurrence of glandular occupation and compromised margins showed the most frequent recurrences [27]. 

Based on the achieved results of dysplasia treatment, the low rates of persistent lesions and recurrence and satisfactory tissue healing, through a safe and non-invasive technique, we propose PDT as a therapeutic option for CIN 1 and 2/3. 

To perform the LEEP surgical procedure 60 days after the non-surgical photodynamic therapy procedure presents several advantages, such as reducing the size of the cervix piece to be removed by LEEP once one or two PDT sessions decrease the CIN size. In addition, PDT has a known action in reducing viral load and when associated with less deep LEEP, will decrease the chances of recurrence, and help to heal the cervix [28,29,30]. 

PDT for CIN lesions can be performed at the ambulatory level, which entails economy for the public health system, and faster treatment of patients who already have an injury to the cervix, even if it is only of a low grade. Especially considering the concept of treatment indication and not only lesion monitoring, there is a psychological gain for the patient, since there will not be the stress involved of knowing she has a lesion that may or may not evolve into a potentially malignant disorder or even to cervical cancer.

Even with three vaccines (Gardasil^®^ and Gardasil^®^9, from Merck & Co., Inc., Kenilworth, New Jersey, and Cervarix^®^, from GlaxoSmithKline Biologicals, Middlesex, United Kingdom), FDA approved and clinically available for protection against the four types of HPV (types 06, 11, 16, and 18) which cause most cervical cancers, they are not effective at treating established HPV infections or disease caused by HPV. Another point is that these vaccines do not protect against all types of HPV that cause cervical cancer, which is why vaccinated women should still be screened for cervical cancer [6]. Therefore, given the established infection, these results indicate PDT as an alternative treatment, especially for those patients who likely will not return to the doctor, ensuring that there will be care for these lesions, reducing the chance that there will be an increase in the degree and avoiding progression, including a possible cervical cancer.

## 4. Materials and Methods

### 4.1. Patient Enrollment

Patients with confirmed diagnosis of cervical intraepithelial neoplasia (CIN) grade 1 and grades 2/3 by histopathology, bearing or not the HPV virus or HIV, were selected and invited to participate in this study. The patient inclusion was consecutive, non-random, and decided by the clinicians involved in the research. 

Fifty-six patients with CIN 1 were treated between April 2013 and October 2015, and monitored up to July 2017. A placebo group formed by 14 patients received only light (*n* = 8) or only topical methyl aminolevulinate cream (*n* = 6), with the same parameters for CIN 1 treatment, and these patients were monitored during the same period. Ten patients with CIN 2/3 were treated between April 2015 and September 2016, and monitored up to December 2018. Written informed consent was obtained following approval by the Human Medical Ethics Committee (CEP 827.010, April 2013). 

### 4.2. Application of MAL Cream

The patient was positioned in a gynecological bed and 2 g of cream containing 20% (w/w) of methyl aminolevulinate (MAL) was delivered at the vagina using a needleless syringe and a tampon was used to keep the cream in place for one hour for CIN 1 or the placebo group, and three hours for CIN 2/3. This cream containing the pro-drug MAL (PDTPharma, Cravinhos-SP, Brazil) penetrates the cells and into the mitochondria, inducing the formation of the photosensitizer protoporphyrin IX (PpIX) locally. 

### 4.3. Fluorescence Images

After one or three hours of cream incubation, the patient was once again placed on the gynecological bed, the tampon was removed, and the excess of cream in the cervix was cleaned using cotton or gauze. The equipment used in this study is a device named “CerCa 150 System^^®^^” produced by MMOptics (Sao Carlos, Sao Paulo, Brazil) in collaboration with the Johns Hopkins University, Rockville, MD, USA. The superficial PpIX production was visualized by a tip with a laser beam with excitation at 405 nm ± 10 nm, and maximum output power of 50 mW ± 20%. The dichroic mirror and specific filters facilitate the visualization of green (mucosa) and red (PpIX) fluorescence. The images were obtained and captured by a portable device (a smartphone Iphone Apple 5 S or 6) connected at the visor probe. 

### 4.4. Photodynamic Therapy

After the fluorescence visualization, the treatment probe with 630 nm LEDs (light-emitting diodes) was positioned for a uniform irradiation of the entire cervix. The irradiation parameters were: irradiance of 80 mW/cm^2^ for 21 min, delivering a total dose of 100.8 J/cm^2^ for patients with CIN 1; irradiance of 120 mW/cm^2^ for 25 min, delivering a total dose of 180 J/cm^2^ for patients with CIN 2/3. After PDT, another fluorescence detection is performed to record the PpIX consumption. The patients with CIN 2/3 received two applications of PDT, one week apart. Sixty days after the second PDT, a less profound loop electrosurgical excision procedure (LEEP) was performed to remove part of the affected cervix which was utilized for histopathological analysis. 

### 4.5. Follow-Up

The patients with CIN 1 were followed at 30 and 90 days after PDT, and by two other long-term follow-ups of one and two years after the treatment. The patients with CIN 2/3 were followed at 60 days after LEEP, and by two other long-term follow-ups at one and two years after the treatment. The clinical evaluation was performed by colposcopy, autofluorescence visualization, and Pap test for all the patients. For the clinical evaluation, a simple microscope was positioned on the cervix and a camera was attached to the microscope and visual inspection was performed before and after the colposcopy. In case of a positive for CIN at the cytology, a biopsy was performed for histopathological analysis. 

## 5. Conclusions

PDT is considered a very attractive technique for the treatment of malignant lesions at an early stage, especially for developing countries. The development of the CerCa 150 System^®^ device and the use of a Brazilian medication containing 20% MAL enables the treatment of these patients for free in the public health system. Another huge multicenter study with over 250 patients with CIN 2/3 is ongoing, showing that topical MAL–PDT has decreased the high-risk HPV load over than 76%, leading us to conclude that it is a promising technique which may be used after the onset of low-grade lesions, or even in cases of more extensive lesions. 

## 6. Patents

United States patent number 9,550,072, granted on January 24, 2017 with MMOptics, LTDA., São Carlos, SP, Brazil as the assignee. 

## Figures and Tables

**Table 1:** Summary of results from patients treated with photodynamic therapy (PDT) for cervical intraepithelial neoplasia (CIN) 1 and 2/3.

**CIN 1**				
**Results**	**General Response of PDT**	**Patients**	**%**	**%**
No dysplasia – 1 year follow up	Positive	7	12.5	75
No dysplasia – 2 years follow up		35	62.5	
CIN 1 remained	Negative	3	5.4	23.2
Progression to CIN 2		5	8.9	
Recurrence		5	8.9	
No follow-up		1	1.8	1.8
**Total**		**56**	**100**	**100**
**CIN 2/3**				
**Results**	**General Response of PDT**	**Patients**	**%**	**%**
No dysplasia – 60 days follow up	Positive	2	20	40
CIN 1 remained		2	20	
CIN 2/3	Negative	6	60	60
**Total**		**10**	**100**	**100**
No dysplasia –1 year follow up	Positive	3	30	90
No dysplasia – 2 years follow up		6	60	
Negative	Negative	1	10	10
**Total**		**10**	**100**	**100**

## References

[1] Saraiya M., Unger E.R., Thompson T.D., Lynch C.F., Hernandez B.Y., Lyu C.W., Steinau M., Watson M., Wilkinson E.J., Hopenhayn C. (2015). US assessment of HPV Types in cancers: Implications for current and 9-valent HPV vaccines. J. Natl. Cancer Inst..

[2] Jemal A., Bray F., Ferlay J. (1999). Global Cancer Statistics: 2011. CA Cancer J. Clin..

[3] Louvanto K., Rintala M.A., Syrjänen K.J., Grénman S.E., Syrjänen S.M. (2010). Genotype-Specific Persistence of Genital Human Papillomavirus (HPV) Infections in Women Followed for 6 Years in the Finnish Family HPV Study. J. Infect. Dis..

[4] Soergel P., Loehr-Schulz R., Hillemanns M., Landwehr S., Makowski L., Hillemanns P. (2010). Effects of photodynamic therapy using topical applied hexylaminolevulinate and methylaminolevulinate upon the integrity of cervical epithelium. Lasers Surg. Med..

[5] Bosch F., Lorincz A., Munoz N., Meijer C., Shah K. (2002). The causal relation between human papillomavirus and cervical cancer. J. Clin. Pathol..

[6] American Cancer S, American Cancer Society (2017). Cancer Facts & Figures. Cancer Facts Fig 2015. https://www.cancer.org/research/cancer-facts-statistics/all-cancer-facts-figures/cancer-facts-figures-2017.html.

[7] Estimativa 2018: Incidência de câncer no Brasil/Instituto Nacional de Câncer, De JAG da SC de P e V– R, Janeiro: INCA 2017. Incidência de Câncer no Brasil-Estimativa 2018. http://www.icabdf.com.br/wp-content/uploads/2017/10/Estimativas_INCA.pdf.

[8] Ho G.Y., Bierman R., Beardsley L., Chang C.J., Burk R.D. (1998). Natural History of Cervicovaginal Papillomavirus Infection in Young Women. N. Engl. J. Med. Feb..

[9] Sadler L., Saftlas A., Wang W., Exeter M., Whittaker J., McCowan L. (2004). Treatment for cervical intraepithelial neoplasia and risk of preterm delivery. JAMA.

[10] Kyrgiou M., Koliopoulos G., Martin-Hirsch P., Arbyn M., Prendiville W., Paraskevaidis E. (2006). Obstetric outcomes after conservative treatment for intraepithelial or early invasive cervical lesions: Systematic review and meta-analysis. Lancet.

[11] Henderson W.B., Dougherty J. (1992). How does therapy work?. Photochem Photobiol..

[12] Hillemanns P., Petry K.U., Soergel P., Collinet P., Ardaens K., Gallwas J., Luyten A., Dannecker C. (2014). Efficacy and safety of hexaminolevulinate photodynamic therapy in patients with low-grade cervical intraepithelial neoplasia. Lasers. Surg. Med..

[13] Hillemanns P., Einstein M.H., Iversen O.E. (2015). Topical hexaminolevulinate photodynamic therapy for the treatment of persistent human papilloma virus infections and cervical intraepithelial neoplasia. Expert Opin. Investig. Drugs.

[14] Van Pachterbeke C., Bucella D., Rozenberg S., Manigart Y., Gilles C., Larsimont D., Vanden Houte K., Reynders M., Snoeck R., Bossens M. (2009). Topical treatment of CIN 2+ by cidofovir: Results of a phase II, double-blind, prospective, placebo-controlled study. Gynecol. Oncol..

[15] Zhang W., Zhang A., Sun W., Yue Y., Li H. (2018). Efficacy and safety of photodynamic therapy for cervical intraepithelial neoplasia and human papilloma virus infection. Medicine (Baltimore).

[16] Inada N.M., da Costa M.M., Guimarães O.C., da Silva Ribeiro E., Kurachi C., Quintana S.M., Lombardi W., Bagnato V.S. (2012). Photodiagnosis and treatment of condyloma acuminatum using 5-aminolevulinic acid and homemade devices. Photodiagn. Photodyn. Ther..

[17] Inada N.M., Lombardi W., Leite M.F.M., Trujillo J.R., Kurachi C., Bagnato V.S. Photodynamic therapy of cervical intraepithelial neoplasia. http://proceedings.spiedigitallibrary.org/proceeding.aspx?doi=10.1117/12.2040004.

[18] Ramirez D.P., Kurachi C., Inada N.M., Moriyama L.T., Salvio A.G., Vollet Filho J.D., Pires L., Buzzá H.H., de Andrade C.T., Greco C. (2014). Experience and BCC subtypes as determinants of MAL-PDT response: Preliminary results of a national Brazilian project. Photodiagn. Photodyn. Ther..

[19] Campbell C.L., Brown C.T.A., Wood K., Salvio A.G., Inada N.M., Bagnato V.S., Moseley H. (2017). A quantitative study of in vivo protoporphyrin IX fluorescence build up during occlusive treatment phases. Photodiagn. Photodyn. Ther..

[20] Blanco K.C., Moriyama L.T., Inada N.M., Sálvio A.G., Menezes P.F., Leite E.J., Kurachi C., Bagnato V.S. (2015). Fluorescence guided PDT for optimization of the outcome of skin cancer treatment. Front. Phys..

[21] Ostör A. (1993). Natural history of cervical intraepithelial neoplasia: A critical review. Int. J. Gynecol. Pathol..

[22] Tao X.H., Guan Y., Shao D., Xue W., Ye F.S., Wang M., He M.H. (2014). Efficacy and safety of photodynamic therapy for cervical intraepithelial neoplasia: A systemic review. Photodiagn. Photodyn. Ther..

[23] Nene B.M., Hiremath P.S., Kane S., Fayette J.M., Shastri S.S., Sankaranarayanan R. (2008). Effectiveness, safety, and acceptability of cryotherapy by midwives for cervical intraepithelial neoplasia in Maharashtra, India. Int. J. Gynecol. Obstet..

[24] Barnett A.A., Haller J.C., Cairnduff F., Lane G., Brown S.B., Roberts D.J. (2003). A randomised, double-blind, placebo-controlled trial of photodynamic therapy using 5-aminolaevulinic acid for the treatment of cervical intraepithelial neoplasia. Int. J. Cancer.

[25] Choi M.C., Jung S.G., Park H., Lee S.Y., Lee C., Hwang Y.Y., Kim S.J. (2013). Photodynamic therapy for management of cervical intraepithelial neoplasia II and III in young patients and obstetric outcomes. Lasers Surg. Med..

[26] Zhu M., He Y., Baak J.P., Zhou X., Qu Y., Sui L., Feng W., Wang Q. (2015). Factors that influence persistence or recurrence of high-grade squamous intraepithelial lesion with positive margins after the loop electrosurgical excision procedure: A retrospective study. BMC Cancer.

[27] Lili E., Chatzistamatiou K., Kalpaktsidou-Vakiani A., Moysiadis T., Agorastos T. (2018). Low recurrence rate of high-grade cervical intraepithelial neoplasia after successful excision and routine colposcopy during follow-up. Medicine.

[28] De Miranda Lima M.I., Melo V.H., Tafuri A., Labanca Â.C., de Miranda Lima L. (2007). Fatores de risco de recidiva de lesões intra-epiteliais cervicais após conização por cirurgia de alta freqüência em mulheres portadoras e não portadoras do vírus da imunodeficiência humana. Rev. Bras. Ginecol. e Obs..

[29] Fu Y., Bao Y., Hui Y., Gao X., Yang M., Chang J. (2016). Topical photodynamic therapy with 5-aminolevulinic acid for cervical high-risk HPV infection. Photodiagn. Photodyn. Ther..

[30] Xie J., Wang S., Li Z., Ao C., Wang J., Wang L., Peng X., Zeng K. (2019). 5-aminolevulinic acid photodynamic therapy reduces HPV viral load via autophagy and apoptosis by modulating Ras/Raf/MEK/ERK and PI3K/AKT pathways in HeLa cells. J. Photochem. Photobiol. B Biol..

